# Endothelial stress as reflected by EASIX predicts cardiovascular morbidity and mortality: Insights from a nationally representative cohort

**DOI:** 10.1016/j.ahjo.2026.100770

**Published:** 2026-03-25

**Authors:** Yu Xia, Mei Wang

**Affiliations:** a Department of Burn and Trauma Medicine, First Naval Hospital, Southern Theater Command, Zhanjiang, China; b Department of Emergency Medicine, First Affiliated Hospital, Anhui Medical University, Hefei, China

**Keywords:** Cardiovascular diseases, Endothelial activation and stress index, Endothelial dysfunction, NHANES, Mortality

## Abstract

**Background:**

Endothelial dysfunction (ED) is a key pathophysiological mechanism in cardiovascular diseases (CVD). The Endothelial Activation and Stress Index (EASIX), derived from routine laboratory markers, has been proposed as a surrogate indicator of endothelial stress. However, its relevance to CVD burden and long-term outcomes in the general population remains uncertain.

**Methods:**

We analyzed data from 38,713 adults aged ≥20 years enrolled in the U.S. National Health and Nutrition Examination Survey (NHANES, 1999–2018), representing approximately 1.7 billion individuals after weighting. EASIX was calculated as [LDH (U/L) × creatinine (mg/dL)] / platelet count (10^9^/L) and log₂-transformed. Weighted logistic regression assessed the association between EASIX and CVD prevalence, while weighted Cox models examined its relationship with all-cause (ACM) and cardiovascular mortality (CVM). Restricted cubic spline analyses evaluated potential nonlinear trends, and subgroup analyses tested effect modification.

**Results:**

Among 38,713 participants, 4131 had CVD. Higher EASIX values were independently associated with greater CVD prevalence (adjusted OR = 1.43, 95% CI: 1.34–1.53). Each log₂ increase in EASIX corresponded to higher risks of ACM (HR = 1.39, 95% CI: 1.28–1.51) and CVM (HR = 1.54, 95% CI: 1.38–1.72). Individuals in the top EASIX quartile exhibited more than double the mortality risks compared with those in the lowest quartile. Associations were nonlinear and consistent across most subgroups.

**Conclusions:**

EASIX demonstrated independent, nonlinear associations with both CVD prevalence and long-term mortality. These findings highlight EASIX as a practical, cost-efficient biomarker for cardiovascular risk stratification in population settings.

## Highlights

• EASIX independently predicts cardiovascular disease prevalence in U.S. adults.
• Elevated EASIX levels are linked to higher all-cause and cardiovascular mortality.
• Nonlinear associations highlight EASIX's dynamic role in vascular pathophysiology.
• Results remained robust after multivariable adjustment and across key subgroups.
• EASIX may serve as a simple, accessible biomarker for cardiovascular risk assessment.

## 1 Introduction

Cardiovascular diseases (CVD), such as ischemic heart disease (IHD) and stroke, remain the leading causes of global mortality and disability. Between 1990 and 2019, the global incidence of CVD nearly doubled, increasing from around 271 million to 523 million cases, with annual CVD-related deaths rising from 12.1 million to 18.6 million in the same timeframe [1], [2]. Early detection of modifiable risk factors is crucial for enhancing prevention strategies and reducing disease-related morbidity and mortality, given the sustained increase.

The vascular endothelium, a single cell layer lining blood vessels, functions as a dynamic regulator of vascular balance, beyond just being a structural barrier. It actively modulates vascular tone, inflammatory responses, oxidative balance, and hemostatic function [3]. Endothelial dysfunction (ED) arises from disrupted endothelial homeostasis, marked by impaired signaling among endothelial cells, leukocytes, and platelets, resulting in increased inflammation and thrombogenic activity. This dysfunction plays a crucial role in the onset of hypertension and related cardiovascular issues [4].

The Endothelial Activation and Stress Index (EASIX), calculated using lactate dehydrogenase(LDH), creatinine, and platelet count, has emerged as a useful measure of ED. Originally developed as a prognostic tool for predicting outcomes in acute graft-versus-host disease cases [5], EASIX was subsequently validated for mortality in allogeneic hematopoietic stem cell transplantation patients [6]. Its prognostic utility has since been extended to a range of clinical conditions, including small-cell lung carcinoma [7], multiple myeloma [8], COVID-19 [9], and sepsis [10]. In the cardiovascular domain, elevated EASIX has been linked to mortality in specific conditions, including hypertension [11], acute myocardial infarction [12], coronary artery disease [13] and atrial fibrillation within intensive care units [14]. Yet, these prognostic assessments are predominantly confined to narrow clinical niches or single-center inpatient cohorts, rendering them susceptible to selection bias, limited sample sizes, and highly variable follow-up durations. A recent population-based assessment drawing on the National Health and Nutrition Examination Survey (NHANES) registry highlighted a direct correlation between amplified EASIX values and a greater burden of specific cardiovascular phenotypes across American adults [15]. Nevertheless, while this offers a valuable epidemiological snapshot of disease frequency, the investigation was fundamentally constrained by its inability to track long-term survival trajectories.

Consequently, a fundamental evidence gap persists: it remains undetermined whether the prognostic utility of EASIX extends beyond isolated clinical subgroups to capture long-term mortality risk across a broader, heterogeneous population of patients with established CVD. To address this limitation, we leveraged comprehensive data from the nationally representative NHANES cohort. Specifically, our research was structured around a dual mandate. First, we sought to quantify the cross-sectional correlation between EASIX scores and overall CVD frequency across the general demographic. Second, we prospectively examined how well this index forecasts long-term survival trajectories—specifically all-cause (ACM) and cardiovascular mortality (CVM)—in the sub-cohort of individuals harboring pre-existing cardiovascular ailments. By analyzing an extended observation window exceeding 140 months, this study seeks to validate EASIX as a unified, practical biomarker for long-term cardiovascular risk stratification, while simultaneously defining its clinical boundary conditions in complex metabolic states.

## 2 Methods

### 2.1 Study population

This cross-sectional study analyzed publicly accessible data from the NHANES, a nationally representative multistage probability survey by the U.S. CDC. NHANES integrates household interviews, standardized physical examinations, and laboratory measurements collected in Mobile Examination Centers (MECs) to evaluate the health and nutritional status of the U.S. population. The National Center for Health Statistics (NCHS) Research Ethics Review Board approved the survey protocol, and participants gave written informed consent.

**Fig. 1:**
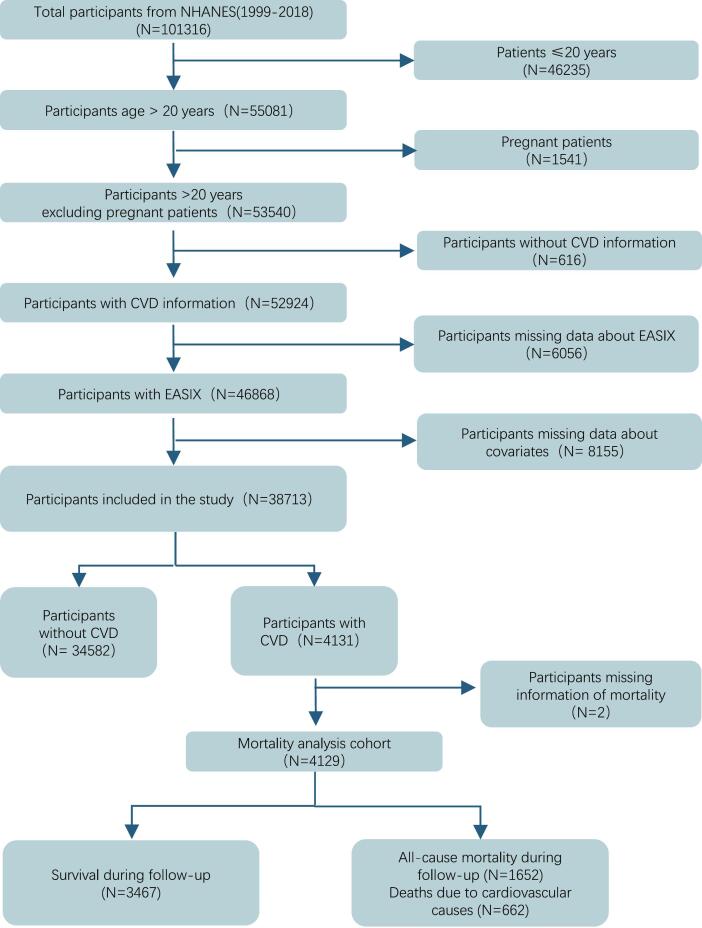
Selection flowchart of participants from NHANES 1999–2018.

Data from ten consecutive NHANES cycles (1999–2018) were combined, encompassing 101,316 individuals. Participants were excluded if they were under 20 years old, pregnant, lacked CVD status information, had incomplete laboratory data for EASIX calculation, or had missing key covariate data for model adjustment. After these exclusions, 38,713 participants were retained for the prevalence analysis, among whom 4131 were identified with CVD. For mortality analyses, two participants were excluded due to missing follow-up data, resulting in a final sample of 4129 individuals with confirmed CVD.(Fig. 1).

### 2.2 Exposure and outcome definition

The primary exposure in this analysis was the EASIX, derived from routinely available laboratory parameters. Specifically, EASIX was calculated as: EASIX = [LDH (U/L) × Creatinine (mg/dL)] / Platelet count (10^9^/L). To normalize distribution and reduce skewness, EASIX values were log2-transformed prior to statistical modeling, consistent with prior research [6]. Blood samples used to calculate EASIX were obtained during the NHANES MEC visit, where laboratory measurements were performed once for each participant as part of the survey examination.

CVD status was determined based on self-reported physician diagnoses. Participants were classified as having CVD if they affirmed having ever been told by a health professional that they had one or more of the following conditions: congestive heart failure (CHF), coronary heart disease (CHD), angina pectoris, myocardial infarction (MI), or stroke. This composite definition captures a broad spectrum of major cardiovascular disorders commonly used in NHANES-based epidemiologic studies.

Mortality endpoints included both ACM and CVM. ACM encompassed death from any cause, while CVM was defined as death attributed to cardiovascular or cerebrovascular conditions, based on ICD-10 codes I00–I09, I11, I13, I20–I51, and I60–I69. Mortality status and cause of death were determined using data from the National Death Index (NDI), linked with NHANES participants through December 31, 2019. Survival time was calculated from the date of the NHANES examination to the date of death or the end of follow-up.

### 2.3 Covariate assessment

Covariates incorporated into the analyses encompassed sociodemographic characteristics, behavioral factors, and medical history. The demographic variables comprised age, gender, race/ethnicity, education level, and the family income-to-poverty ratio (FIPR). The behavioral variables considered were smoking status, alcohol consumption, and body mass index (BMI). Clinical comorbidities comprised self-reported diagnoses of hypertension, diabetes mellitus, hyperlipidemia, chronic kidney disease (CKD), and metabolic syndrome (MetS). Operational definitions and coding details for these variables are provided in Supplementary Table S1.

### 2.4 Statistical analysis

Statistical analyses incorporated NHANES’ complex multistage sampling design. MEC examination weights were applied to generate nationally representative estimates and to adjust for unequal probabilities of selection, nonresponse, and post-stratification. Continuous variables were represented by medians and interquartile ranges (Q1–Q3), with between-group differences assessed via design-based Kruskal–Wallis tests incorporating the Rao & Scott correction. Categorical variables were displayed as unweighted frequencies and percentages, and analyzed using Pearson's chi-squared test with the Rao & Scott adjustment.

Weighted logistic regression was used to analyze the relationship between EASIX and CVD prevalence, with EASIX considered as both a continuous variable and divided into quartiles. Estimated odds ratios (ORs) with 95% confidence intervals (CIs). Weighted Cox proportional hazards models were applied to assess mortality outcomes (ACM and CVM), incorporating EASIX as both continuous and categorical variables. Adjusted hazard ratios (HRs) with 95% confidence intervals (CIs) were computed for each quartile compared to the lowest quartile (Q1), accounting for all covariates in the final model. Potential nonlinear associations between EASIX and each outcome were explored using restricted cubic spline (RCS) functions with four knots, and the significance of nonlinearity was formally tested. Kaplan–Meier survival curves were used to illustrate the cumulative incidence of ACM and CVM across EASIX quartiles, with survival differences assessed via the log-rank test. Subgroup analyses were performed to investigate potential effect modification based on demographic and clinical characteristics. Interaction terms between EASIX and relevant covariates were incorporated into the regression models, and results were summarized graphically using forest plots to depict heterogeneity across subpopulations. Analyses were conducted using a two-tailed approach with statistical significance defined as *P* < 0.05.The analyses utilized R software (version 4.4.2) and the survey package to properly incorporate sampling weights and survey design.

## 3 Results

### 3.1 Baseline characteristics of participants

The final weighted analysis included 38,713 participants, representing approximately 1.7 billion U.S. adults. A total of 4131 individuals were identified with CVD, corresponding to an estimated 13.9 million cases across the nation (Table 1). Participants with CVD had a median age of 66 years, which was older than the 45 years median age of those without CVD. The CVD group also had a slightly higher proportion of males (53.5%) relative to females (46.4%).Racial distribution revealed that non-Hispanic Whites comprised a greater percentage of the CVD population (76.4%) compared with the non-CVD group (69.1%), while Mexican American and other Hispanic participants were underrepresented among CVD cases. Socioeconomic disparities were evident as individuals with CVD were more likely to have lower educational attainment (less than high school, 24.6% compared to 15.0%) and income levels (FIPR <1.30, 27.4% compared to 20.1%).The prevalence of metabolic and chronic comorbidities, including hypertension, diabetes, hyperlipidemia, CKD, and MetS, was markedly higher among participants with CVD. These findings underscore the substantial clustering of cardiometabolic disorders among those affected by CVD.

### 3.2 Association of EASIX and prevalence of CVD

**Fig. 2:**
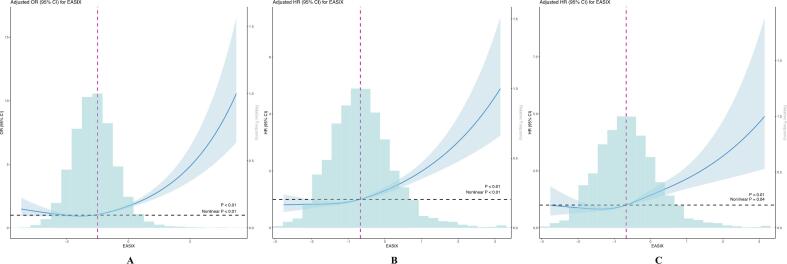
Restricted cubic spline regression analysis between the EASIX and (A) the prevalence, (B) ACM, and (C) CVM of CVD. 4-knotted RCS model adjusted for age, gender, race, FPIR, marital status, education level, smoking, drinking, BMI, diabetes, hypertension, hyperlipidemia, CKD, MetS. EASIX values shown on the x-axis represent log₂-transformed EASIX, which was used in all statistical analyses to reduce skewness of the distribution.

Weighted logistic regression analyses were performed using EASIX as both a continuous variable and in quartile categories. As detailed in Table 2, a significant positive relationship was observed between EASIX and CVD prevalence, evident before adjustment (odds ratio [OR] = 2.57; 95% confidence interval [CI], 2.40–2.76) and persisting after comprehensive adjustment for confounders (OR = 1.43; 95% CI, 1.34–1.53). In the fully adjusted model, participants in the highest quartile (Q4) had a 53% higher odds of CVD than those in the lowest quartile (Q1) (OR = 1.53; 95% CI, 1.32–1.77; *P* < 0.001), with a significant linear trend observed across quartiles (P for trend <0.001). Furthermore, RCS analysis with four knots confirmed a robust association between EASIX and CVD prevalence after adjustment (*P* < 0.01), revealing a nonlinear dose-response relationship (P for nonlinear <0.01), as depicted in Fig. 2A.

**Table 2:** presents a weighted logistic regression analysis examining the relationship between EASIX and CVD prevalence.

Outcome	Models					
	1		2		3	
	OR (95%CI)	*P* value	OR (95%CI)	*P* value	OR (95%CI)	*P* value
EASIX(continuous)	2.57 (2.40–2.76)	<0.001	1.54 (1.44–1.65)	<0.001	1.43 (1.34–1.53)	<0.001
EASIX(quartiles)						
Q1	Reference		Reference		Reference	
Q2	1.16 (1.01–1.33)	0.034	0.83 (0.72–0.97)	0.018	0.89 (0.76–1.04)	0.131
Q3	1.67 (1.48–1.89)	<0.001	0.91 (0.79–1.05)	0.181	1.00 (0.86–1.15)	0.964
Q4	4.26 (3.78–4.80)	<0.001	1.51 (1.31–1.74)	<0.001	1.53 (1.32–1.77)	<0.001
*P* for trend		<0.001		<0.001		<0.001

Model 1: Unadjusted; Model 2: Adjusted age, gender, and race; Model 3: Further adjusted smoking, drinking, BMI, hypertension, diabetes, hyperlipidemia, CKD and MetS.

### 3.3 Correlation between EASIX and mortality in CVD patients

**Fig. 3:**
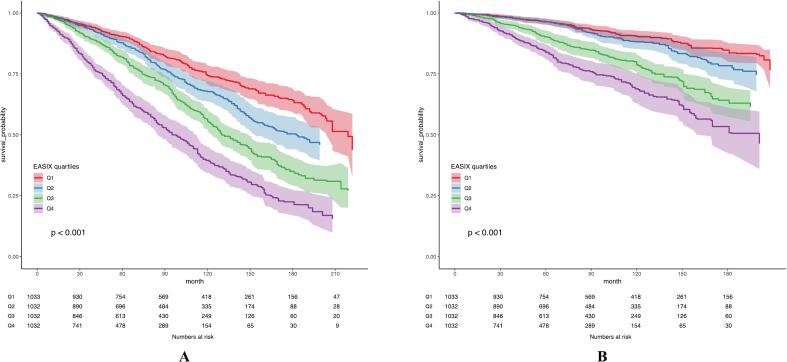
Kaplan-Meier plots for (A) ACM and (B) CVM in individuals with CVD.

Over a median follow-up of 142 months (95% CI 134–147), 1652 out of 4129 participants with CVD died, resulting in an ACM rate of 40.01%.Among these, 662 deaths (16.03%) were attributed to cardiovascular causes. Baseline characteristics by EASIX quartiles are presented in Table 3.In weighted Cox regression analyses, higher EASIX was independently associated with increased risk of both ACM and CVM. After adjusting for confounders, a one-unit increase in EASIX correlated with a 39% rise in ACM risk (HR = 1.39; 95% CI, 1.28–1.51) and a 54% rise in CVM risk (HR = 1.54; 95% CI, 1.38–1.72) in CVD patients. Compared to Q1, participants in Q3 had a 52% higher risk of ACM (HR = 1.52, 95% CI 1.24–1.85), and those in Q4 had a 132% increased risk (HR = 2.32, 95% CI 1.88–2.87). For CVM, the corresponding HRs were 1.68 (95% CI: 1.26–2.25) for Q3 and 2.37 (95% CI: 1.76–3.20) for Q4 (Table 4).Kaplan–Meier survival analyses corroborated these results, demonstrating significantly worse survival for participants in higher EASIX quartiles (Q4) for both ACM and CVM (Fig. 3). Moreover, RCS analyses indicated a nonlinear association between EASIX and ACM (P for nonlinear <0.01, Fig. 2B) as well as CVM (P for nonlinear = 0.04, Fig. 2C).

**Table 4:** presents a weighted Cox regression analysis examining the relationship between EASIX and mortality outcomes among participants with CVD.

Outcome	Models					
	1		2		3	
	HR (95%CI)	*P* value	HR (95%CI)	*P* value	HR (95%CI)	*P* value
ACM						
EASIX(continuous)	1.68 (1.58–1.79)	<0.001	1.41 (1.32–1.52)	<0.001	1.39 (1.28–1.51)	<0.001

EASIX(quartiles)						
Q1	Reference		Reference		Reference	
Q2	1.36 (1.12–1.66)	<0.01	1.12 (0.92–1.36)	0.276	1.17 (0.96–1.43)	0.122
Q3	2.02 (1.70–2.42)	<0.001	1.49 (1.23–1.81)	<0.001	1.52 (1.24–1.85)	<0.001
Q4	3.39 (2.88–3.98)	<0.001	2.36 (1.95–2.85)	<0.001	2.32 (1.88–2.87)	<0.001
*P* for trend		<0.001		<0.001		<0.001
CVM						
EASIX(continuous)	1.79 (1.66–1.94)	<0.001	1.64 (1.49–1.80)	<0.001	1.54 (1.38–1.72)	<0.001

EASIX(quartiles)						
Q1	Reference		Reference		Reference	
Q2	1.26 (0.95–1.66)	0.106	0.99 (0.75–1.31)	0.955	1.02 (0.77–1.36)	0.892
Q3	2.46 (1.90–3.18)	<0.001	1.71 (1.30–2.25)	<0.001	1.68 (1.26–2.25)	<0.001
Q4	3.98 (3.11–5.09)	<0.001	2.59 (1.98–3.40)	<0.001	2.37 (1.76–3.20)	<0.001
*P* for trend		<0.001		<0.001		<0.001

Model 1: Unadjusted; Model 2: Adjusted age, gender, and race; Model 3: Further adjusted smoking, drinking, BMI, hypertension, diabetes, hyperlipidemia, CKD and MetS.

**Table 1:** Baseline characteristics of study population.

	Overall	By CVD Status		
Characteristic	Overall, *N* = 38,713 (100%)	No, *N* = 34,582 (92%)	Yes, *N* = 4131 (8%)	p-value^1^
Weighted participants, n	167,953,802	154,031,605	13,922,196	NA
Age, years, median, IQR	46.00 (33.00,59.00)	45.00 (32.00,57.00)	66.00 (56.00,76.00)	<0.001
Gender, n (%)				<0.001
Male	19,183 (48.84%)	16,842 (48.42%)	2341 (53.53%)	
Female	19,530 (51.16%)	17,740 (51.58%)	1790 (46.47%)	
Race, n (%)				<0.001
Mexican American	6289 (7.92%)	5890 (8.30%)	399 (3.73%)	
Other Hispanic	3130 (5.11%)	2882 (5.29%)	248 (3.13%)	
Non-Hispanic White	17,715 (69.68%)	15,342 (69.07%)	2373 (76.36%)	
Non-Hispanic Black	7880 (10.44%)	7006 (10.39%)	874 (10.99%)	
Other or multiracial	3699 (6.85%)	3462 (6.94%)	237 (5.78%)	
Education level, n (%)				<0.001
Less than high school	9605 (15.81%)	8220 (15.02%)	1385 (24.64%)	
High school or equivalent	8979 (23.72%)	7941 (23.37%)	1038 (27.57%)	
Above high school	20,129 (60.47%)	18,421 (61.61%)	1708 (47.79%)	
Marital status, n (%)				<0.001
Coupled	23,448 (64.45%)	21,134 (64.82%)	2314 (60.45%)	
Single or separated	15,265 (35.55%)	13,448 (35.18%)	1817 (39.55%)	
FIPR, n (%)				<0.001
<1.30	11,760 (20.72%)	10,247 (20.12%)	1513 (27.41%)	
1.30–3.50	14,807 (35.98%)	13,112 (35.44%)	1695 (41.85%)	
>3.50	12,146 (43.30%)	11,223 (44.44%)	923 (30.74%)	
Smoking, n (%)				<0.001
Never	20,890 (53.85%)	19,302 (55.28%)	1588 (38.04%)	
Former	9620 (24.88%)	7927 (23.51%)	1693 (39.98%)	
Now	8203 (21.27%)	7353 (21.20%)	850 (21.99%)	
Drinking, n (%)				<0.001
Mild	24,920 (60.44%)	21,646 (58.93%)	3274 (77.10%)	
Moderate	7602 (22.16%)	7185 (23.17%)	417 (10.97%)	
Heavy	6191 (17.40%)	5751 (17.90%)	440 (11.93%)	
BMI, kg/m^2^, median, IQR	27.70 (24.09,32.20)	27.57 (24.00,32.00)	29.40 (25.60,33.95)	<0.001
Diabetes, n (%)	6306 (11.99%)	4724 (9.97%)	1582 (34.31%)	<0.001
Hypertension, n (%)	13,895 (31.46%)	10,864 (27.91%)	3031 (70.80%)	<0.001
Hyperlipidemia, n (%)	24,148 (61.79%)	20,925 (60.14%)	3223 (80.06%)	<0.001
CKD, n (%)	1146 (2.26%)	711 (1.67%)	435 (8.86%)	<0.001
MetS, n (%)	8137 (18.30%)	5884(15.03%)	2253 (54.40%)	<0.001
LDH U/L, median, IQR	128.00 (113.00,146.00)	127.00 (112.00,145.00)	136.00 (120.00,158.00)	<0.001
Creatinine, mg/dL, median, IQR	0.86 (0.72,1.00)	0.85 (0.72,1.00)	0.97 (0.80,1.16)	<0.001
PLT, 10^9^/L, median, IQR	245.00 (209.00,290.00)	247.00 (211.00,291.00)	226.00 (186.00,273.00)	<0.001
EASIX, median, IQR	−1.16 (−1.58,-0.74)	−1.18 (−1.60,-0.78)	−0.75 (−1.27,-0.24)	<0.001
Untransformed EASIX, median, IQR	0.45 (0.33–0.60)	0.44 (0.33–0.58)	0.60 (0.42–0.85)	<0.001

1 Design-based Kruskal Wallis test; Pearson's X^2: Rao & Scott adjustment Abbreviations: BMI, body mass index; CKD, chronic kidney disease; MetS, metabolic syndrome; LDH, lactate dehydrogenase; PLT, platelet count; EASIX, Endothelial Activation and Stress Index; IQR, interquartile range. EASIX was calculated as LDH (U/L) × creatinine (mg/dL) / platelet count (10^9^/L). EASIX values were log₂-transformed for statistical analyses. Untransformed EASIX values are additionally presented for reference.

**Table 3:** Baseline characteristics of CVD participants grouped by EASIX quartiles.

Characteristic	Overall	By EASIX quartiles				
	*N* = 4129	Q1(≤ − 1.57)	Q2(−1.57 to − 1.13)	Q3(−1.13 to − 0.68)	Q4(≥ − 0.68)	p-value^1^
Weighted participants, n	13,916,214	3,887,702	3,716,651	3,300,480	3,011,382	NA
Age, years, median, IQR	66.00 (56.00,76.00)	58.00 (48.00,69.00)	65.00 (56.00,74.00)	70.00 (60.00,78.00)	72.00 (65.00,80.00)	<0.001
Gender, n (%)						<0.001
Male	2340 (53.53%)	344 (32.72%)	560 (52.77%)	692 (64.83%)	744 (68.97%)	
Female	1789 (46.47%)	689 (67.28%)	472 (47.23%)	340 (35.17%)	288 (31.03%)	
Race, n (%)						0.001
Mexican American	399 (3.74%)	154 (5.63%)	103 (3.24%)	73 (3.00%)	69 (2.72%)	
Other Hispanic	248 (3.13%)	81 (4.30%)	66 (2.84%)	44 (2.44%)	57 (2.75%)	
Non-Hispanic White	2372 (76.37%)	562 (74.75%)	599 (77.55%)	611 (75.89%)	600 (77.53%)	
Non-Hispanic Black	873 (10.98%)	174 (9.28%)	210 (10.63%)	229 (11.35%)	260 (13.20%)	
Other or multiracial	237 (5.78%)	62 (6.05%)	54 (5.73%)	75 (7.33%)	46 (3.81%)	
Education level, n (%)						0.2
Less than high school	1384 (24.62%)	375 (26.03%)	342 (23.04%)	347 (25.46%)	320 (23.86%)	
High school or equivalent	1038 (27.58%)	274 (30.22%)	246 (26.28%)	268 (26.84%)	250 (26.58%)	
Above high school	1707 (47.80%)	384 (43.75%)	444 (50.69%)	417 (47.70%)	462 (49.56%)	
Marital status, n (%)						0.031
Coupled	2312 (60.43%)	542 (55.64%)	578 (61.48%)	598 (63.48%)	594 (62.00%)	
Single or separated	1817 (39.57%)	491 (44.36%)	454 (38.52%)	434 (36.52%)	438 (38.00%)	
FIPR, n (%)						0.006
<1.30	1512 (27.40%)	444 (33.69%)	398 (26.69%)	350 (24.89%)	320 (22.90%)	
1.30–3.50	1695 (41.87%)	381 (38.05%)	406 (42.17%)	446 (42.47%)	462 (45.78%)	
>3.50	922 (30.73%)	208 (28.26%)	228 (31.14%)	236 (32.64%)	250 (31.32%)	
Smoking, n (%)						<0.001
Never	1586 (38.01%)	376 (34.76%)	401 (39.24%)	391 (38.38%)	418 (40.28%)	
Former	1693 (39.99%)	344 (31.91%)	396 (38.84%)	457 (42.72%)	496 (48.85%)	
Now	850 (21.99%)	313 (33.33%)	235 (21.91%)	184 (18.89%)	118 (10.87%)	
Drinking, n (%)						0.005
Mild	3272 (77.09%)	777 (73.50%)	798 (75.91%)	832 (79.43%)	865 (80.60%)	
Moderate	417 (10.98%)	132 (13.40%)	114 (11.42%)	105 (11.71%)	66 (6.51%)	
Heavy	440 (11.94%)	124 (13.10%)	120 (12.67%)	95 (8.87%)	101 (12.89%)	
BMI, kg/m^2^, median, IQR	29.39 (25.60,33.92)	29.78 (25.14,34.24)	29.28 (25.50,34.00)	29.08 (26.08,33.70)	29.32 (25.80,33.70)	0.9
Diabetes, n (%)	1581 (34.31%)	373 (30.42%)	354 (30.51%)	393 (35.67%)	461 (42.54%)	<0.001
Hypertension, n (%)	3030 (70.82%)	712 (66.33%)	744 (69.53%)	763 (72.39%)	811 (76.47%)	0.002
Hyperlipidemia, n (%)	3223 (80.10%)	785 (77.88%)	831 (82.51%)	829 (82.32%)	778 (77.56%)	0.018
CKD, n (%)	435 (8.86%)	53 (4.88%)	56 (4.06%)	71 (5.81%)	255 (23.24%)	<0.001
MetS, n (%)	2253 (54.43%)	493 (47.04%)	545 (53.38%)	600 (60.01%)	615 (59.14%)	<0.001
LDH U/L, median, IQR	136.00 (120.00,158.00)	120.00 (107.00,135.00)	132.00 (120.00,149.00)	142.00 (127.00,160.00)	165.00 (143.00,189.00)	<0.001
Creatinine, mg/dL, median, IQR	0.97 (0.80,1.16)	0.79 (0.69,0.90)	0.92 (0.81,1.04)	1.06 (0.94,1.20)	1.30 (1.07,1.61)	<0.001
PLT, 10^9^/L, median, IQR	226.00 (186.00,273.00)	286.00 (247.00,331.00)	235.00 (206.00,266.00)	204.00 (181.00,235.00)	170.00 (144.00,200.00)	<0.001
ACM, n (%)	1652 (34.80%)	307 (26.53%)	365 (29.91%)	442 (39.01%)	538 (46.88%)	<0.001
CVM, n (%)	662 (13.56%)	108 (9.51%)	127 (9.87%)	196 (16.89%)	231 (19.67%)	<0.001

1 Design-based Kruskal Wallis test; Pearson's X^2: Rao & Scott adjustment Abbreviations: BMI, body mass index; CKD, chronic kidney disease; MetS, metabolic syndrome; LDH, lactate dehydrogenase; PLT, platelet count; EASIX, Endothelial Activation and Stress Index; IQR, interquartile range; ACM: all-cause mortality; CVM: cardiovascular mortality. EASIX was calculated as LDH (U/L) × creatinine (mg/dL) / platelet count (10^9^/L). Quartiles were defined according to log₂-transformed EASIX values. The corresponding ranges of untransformed EASIX were: Q1, ≤0.34; Q2, 0.34–0.46; Q3, 0.46–0.62; Q4, ≥0.62.

### 3.4 Analysis of EASIX in relation to CVD prevalence and mortality outcomes

**Fig. 4:**
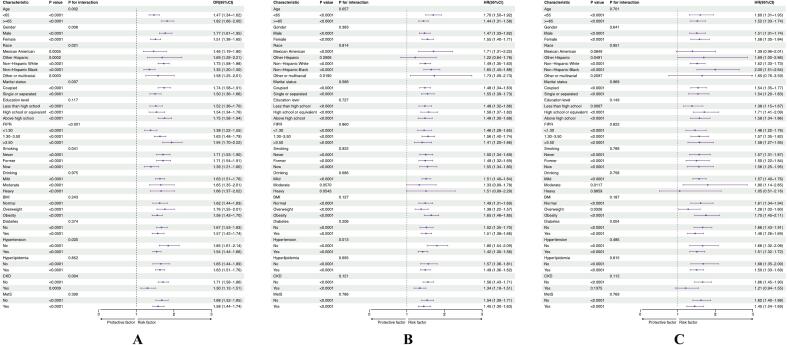
Subgroup analysis as well as interaction test of the interplay between EASIX and (A) the prevalence,(B) ACM, (C) and CVM of CVD. Covariates included age, gender, race, FPIR, marital status, education level, smoking, drinking, BMI, diabetes, hypertension, hyperlipidemia, CKD, MetS.

Subgroup analyses examined the uniformity of associations among various demographic and clinical groups. Overall, EASIX showed positive associations with CVD prevalence, ACM, and CVM across most population strata. However, interaction analyses revealed that the strength of these relationships varied across certain comorbid conditions and demographic factors (Fig. 4).

Notable interactions were identified between EASIX and variables such as age, sex, race, marital status, FIPR, smoking status, hypertension, and CKD concerning CVD prevalence (all P for interaction <0.05), indicating varying risk patterns among subgroups. EASIX maintained a significant association with ACM across most groups; however, this association was weakened among Other Hispanic participants (*P* = 0.2906) and those with moderate (*P* = 0.0570) or heavy alcohol consumption (*P* = 0.0543). Notably, interaction testing indicated a significant modifying effect of hypertension (*P* = 0.013). Regarding CVM, EASIX retained positive associations across most strata; however, results were nonsignificant among Mexican Americans (*P* = 0.0849), Other or Multiracial participants (P = 0.0543), heavy drinkers (*P* = 0.8859), and individuals with CKD (*P* = 0.1375). A statistically significant interaction was identified between EASIX and diabetes (P for interaction = 0.004), suggesting potential effect modification by glycemic status.

Collectively, these results demonstrate that elevated EASIX levels are robustly and nonlinearly related to both CVD prevalence and long-term mortality, though the strength of association may differ depending on metabolic and clinical context.

## 4 Discussion

The vascular endothelium is integral to maintaining vascular homeostasis [16]. Consequently, ED—phenotypically characterized by impaired nitric oxide bioavailability, elevated oxidative stress, and a shift toward pro-inflammatory and prothrombotic states—acts as a primary mechanistic driver in the pathogenesis and progression of CVD [4]. While ED has conventionally been evaluated using non-invasive imaging modalities such as flow-mediated dilation (FMD) [17], the EASIX has recently gained traction as a highly accessible, routine blood-based surrogate for endothelial impairment [11], [12], [14]. Leveraging an extensive, population-level U.S. sample, this study demonstrates that higher EASIX scores directly track with increased CVD prevalence and worse long-term survival. This dose-response relationship proved to be strictly nonlinear and resilient to comprehensive covariate adjustment; notably, the susceptibility to all-cause and cardiovascular death was profoundly magnified in the top EASIX quartile (Q4) when juxtaposed with the lowest (Q1).

The prognostic utility of EASIX across such a broad CVD spectrum lies in how its three parameters collectively capture the pathophysiology of endothelial stress. Elevated LDH acts as a primary indicator of tissue damage and systemic inflammation [18], [19]. As oxidative stress and inflammatory cascades trigger endothelial cell apoptosis or necrosis [3], LDH leaks into the circulation from the injured endothelium, as well as from activated leukocytes and platelets [20]. In parallel, a reduced platelet count flags the physical disruption of the vascular lining. When the endothelium is compromised, the exposure of subendothelial von Willebrand factor (vWF) provokes continuous platelet adhesion and peripheral consumption [21], directly driving the thrombo-inflammatory processes that lead to atherothrombosis. Furthermore, the inclusion of serum creatinine grounds the index in cardiorenal hemodynamics. Since widespread endothelial injury forms the pathogenic basis for renal impairments like acute kidney injury [22] and diabetic nephropathy [23], creatinine levels reflect the downstream microvascular consequences of this systemic vascular deterioration. Ultimately, the EASIX formula does not just list these biomarkers; it mathematically integrates them to amplify the signal of microvascular failure, offering a highly sensitive snapshot of the physiological decline preceding cardiovascular mortality.

While this mechanistic foundation is robust, the actual prognostic accuracy of EASIX in clinical settings is inevitably modulated by a patient's coexisting metabolic burden. Our subgroup analyses revealed significant interactions between EASIX and comorbidities like hypertension and CKD concerning CVD prevalence. This aligns with established biology: hypertension mechanically and biochemically exacerbates endothelial damage via cyclooxygenase-2 (COX-2) overexpression and diminished nitric oxide (NO) availability [24]. Consequently, elevated EASIX levels already correlate with increased mortality in hypertensive populations [11]. Since hypertension acts as a primary catalyst for a cascade of downstream events—spanning stroke, coronary artery disease, heart failure, and sudden cardiac death [25]—this extensive vascular damage naturally amplifies the association between EASIX and CVD. Similarly, ED is a fundamental pathological feature of both acute and chronic kidney injuries [22], [23]. We found the link between EASIX and mortality was markedly attenuated in patients with CKD. In this population, ED is driven by distinct pathways, such as TGF-β-mediated capillary rarefaction [26] and FOXK1-induced metabolic shifts [27]. The blunted predictive power here likely stems from a quantitative “ceiling effect.” Because serum creatinine anchors the EASIX formula, its profound elevation in advanced renal disease severely skews the index's distribution, effectively masking its ability to discriminate isolated cardiovascular events against a backdrop of renal failure. A similar confounding pattern emerged regarding alcohol consumption. While moderate intake offers modest vascular protection [28], excessive drinking actively degrades vascular integrity [29], which likely disrupts the specific pathophysiological signals EASIX relies on, explaining the index's diminished prognostic accuracy for all-cause mortality in heavy drinkers.

Ultimately, ED acts as the shared pathological substrate for a host of chronic metabolic and inflammatory conditions, including diabetes, hypertension, and metabolic syndrome [30], [31]. Therefore, the clinical utility of EASIX is not uniform; it fluctuates based on the specific disease burden and how those overlapping pathologies interact with cardiovascular hemodynamics. Moving forward, highly stratified analyses are required to validate these findings and accurately map the dynamic performance of EASIX across distinct clinical phenotypes.

Beyond its pathophysiological insights, the practical value of EASIX lies in its immediate clinical applicability. Because the index is derived entirely from ubiquitous and inexpensive routine laboratory panels, it effectively circumvents the logistical and financial barriers associated with the specialized equipment or trained sonographers required for assessments like FMD or RH-PAT. In primary care and resource-limited clinical settings, EASIX functions as a highly accessible early warning system. Physicians can leverage baseline scores to pinpoint individuals harboring a high subclinical endothelial burden—patients who are prime candidates for more aggressive, targeted interventions, such as intensive lipid-lowering therapy, antithrombotic optimization, or novel anti-inflammatory agents. Transitioning EASIX from an observational prognostic marker to an actionable clinical tool, however, demands rigorous prospective validation. Randomized controlled trials are now essential to determine whether therapeutic escalation guided by EASIX tangibly alters cardiovascular survival trajectories. Furthermore, longitudinal studies must establish whether dynamic reductions in EASIX scores over time reliably mirror treatment efficacy and arterial plaque stabilization.

This study is bolstered by several methodological strengths, most notably the utilization of a large, nationally representative cohort (NHANES) paired with an extended follow-up period exceeding 140 months. By advancing beyond recent cross-sectional evaluations [15], this robust longitudinal framework substantially enhances the external validity of our findings. While our conclusions are compelling, they are inherently subject to specific structural constraints. Foremost, the epidemiological snapshot used to capture disease frequency, coupled with the observational nature of the mortality follow-up, prevents the establishment of direct causal links. Therefore, the precise temporal mechanics driving the relationship between the EASIX metric and disease progression are not yet fully delineated. Beyond these temporal ambiguities, our analysis must also contend with residual confounding. Even with rigorous survey-weighted covariate adjustments, the absence of high-resolution clinical variables—such as distinct CVD subtypes, symptomatic severity, and longitudinal pharmacological regimens—leaves room for unmeasured modifiers. Compounding this issue is the reliance on self-reported diagnostic histories; categorizing cardiovascular status through personal recall inherently risks misclassification bias, thereby compromising the ultimate precision of our prognostic hazard ratios. Furthermore, the geographic confinement of the NHANES registry to the United States restricts the universal applicability of these results. It remains to be seen how EASIX performs across disparate global cohorts characterized by unique genetic backgrounds, lifestyle paradigms, and healthcare infrastructures. Ultimately, these constraints highlight the immediate need for prospective longitudinal validation across diverse demographic groups and richer clinical datasets, which will be essential for solidifying the clinical value of EASIX in cardiovascular risk assessment.

## 5 Conclusion

In conclusion, our findings reveal a distinct nonlinear relationship between EASIX and both CVD prevalence and long-term mortality in U.S. adults. Higher EASIX values were significantly associated with increased risks of all-cause and cardiovascular mortality, even after adjusting for multiple confounders. These results underscore the potential of EASIX as a practical and economical biomarker reflecting endothelial health and systemic vascular burden. Given its derivation from routine laboratory tests, EASIX could be readily implemented in public health screening and clinical risk stratification to identify individuals at elevated cardiovascular risk. Further prospective studies across varied populations are necessary to confirm these associations, investigate causal mechanisms, and assess the potential of EASIX-guided interventions to enhance cardiovascular outcomes on a broader scale.

## CRediT authorship contribution statement

**Yu Xia:** Writing – original draft, Formal analysis, Data curation, Conceptualization. **Mei Wang:** Writing – review & editing, Supervision, Resources, Methodology, Investigation, Conceptualization.

## Consent for publication

Not applicable.

## Ethical statement

This study was conducted using publicly available, de-identified data from the National Health and Nutrition Examination Survey (NHANES), administered by the U.S. Centers for Disease Control and Prevention (CDC). NHANES protocols were approved by the National Center for Health Statistics Research Ethics Review Board, and all participants provided written informed consent. As this analysis involved secondary use of anonymized public data, no additional ethical approval was required.

## Declarations

Approval for the study was obtained from the relevant ethics committee, and informed consent was secured from all participants.

This study utilized publicly accessible data from the National Health and Nutrition Examination Survey (NHANES), managed by the National Center for Health Statistics (NCHS). The NCHS Research Ethics Review Board approved all NHANES protocols, and informed consent was obtained from participants before data collection. Since this study utilized secondary analysis of de-identified public data, further institutional ethical approval was unnecessary.

## Clinical trial number

Not applicable.

## Funding

None.

## Declaration of competing interest

The authors state there are no conflicts of interest.

## Data Availability

The datasets used in this study are publicly accessible in the NHANES repository, managed by the U.S. CDC. NHANES data and documentation can be accessed at: The study utilizes de-identified data in accordance with NHANES data use policy.

## Appendix A Supplementary data

Supplementary Table S1 Definitions of complex variables and derived markers
