# Supplementary material for: Endothelial stress as reflected by EASIX predicts cardiovascular morbidity and mortality: Insights from a nationally representative cohort
Source: Am Heart J Plus. 2026 Mar 25;65:100770. doi: 10.1016/j.ahjo.2026.100770 (PMC13054611; doi:10.1016/j.ahjo.2026.100770)
Supplement: Supplementary Table S1 — Definitions of complex variables and derived markers [file mmc1.docx]

**Definitions of Complex Variables and Derived Markers**

1. **Definitions of Clinical Conditions**

**Diabetes Mellitus** was defined according to the American Diabetes Association guidelines and NHANES criteria ^[1]^ as the presence of **any** of the following:

1. A self-reported history of diabetes diagnosis;
2. Current use of insulin injections;
3. Current use of oral hypoglycemic medications;
4. Glycohemoglobin (HbA1c) ≥ 6.5%;
5. Fasting plasma glucose (FPG) ≥ 126 mg/dL.

**Hypertension** was defined based on the criteria from the 2024 ESC Guidelines for the management of elevated blood pressure and hypertension, met by any of the following ^[2]^:

1. Systolic blood pressure (SBP) ≥ 140 mmHg;
2. Diastolic blood pressure (DBP) ≥ 90 mmHg;
3. Self-reported use of antihypertensive medication.

**Metabolic Syndrome (MetS)** was diagnosed according to the revised NCEP ATP III criteria ^[3]^, requiring at least **three** of the following five components:

1. **Abdominal obesity**: Waist circumference > 102 cm (40 inches) in men or > 89 cm (35 inches) in women;
2. **Elevated triglycerides (TG)**: ≥ 150 mg/dL (1.7 mmol/L), or on lipid-lowering treatment;
3. **Reduced HDL-C**: < 40 mg/dL (1.0 mmol/L) in men or < 50 mg/dL (1.3 mmol/L) in women, or on lipid-lowering treatment;
4. **Elevated blood pressure**: SBP ≥ 130 mmHg or DBP ≥ 85 mmHg, or on antihypertensive treatment;
5. **Elevated fasting glucose**: FPG ≥ 100 mg/dL (5.6 mmol/L), or on antidiabetic treatment.

**Hyperlipidemia** was defined based on the presence of **any** of the following four lipid abnormalities ^[3]^:

1. Total cholesterol (TC) ≥ 200 mg/dL (5.2 mmol/L);
2. LDL cholesterol (LDL-C) ≥ 130 mg/dL (3.4 mmol/L);
3. HDL cholesterol (HDL-C): < 40 mg/dL (1.0 mmol/L) in men, or < 50 mg/dL (1.3 mmol/L) in women;
4. Triglycerides (TG) ≥ 150 mg/dL (1.7 mmol/L).

**Reference**

[1] Zhao J, Fu S, Chen Q. Association between the serum vitamin D level and prevalence of obesity/abdominal obesity in women with infertility: a cross-sectional study of the National Health and Nutrition Examination Survey data[J]. Gynecol Endocrinol, 2023, 39(1): 2217251.

[2] Mcevoy J W, Mccarthy C P, Bruno R M, et al. 2024 ESC Guidelines for the management of elevated blood pressure and hypertension[J]. Eur Heart J, 2024, 45(38): 3912-4018.

[3] Executive Summary of The Third Report of The National Cholesterol Education Program (NCEP) Expert Panel on Detection, Evaluation, And Treatment of High Blood Cholesterol In Adults (Adult Treatment Panel III)[J]. Jama, 2001, 285(19): 2486-97.
